# Supplementary material for: Neural flip-flops I: Short-term memory
Source: PLoS One. 2024 Mar 15;19(3):e0300534. doi: 10.1371/journal.pone.0300534 (PMC10942071; doi:10.1371/journal.pone.0300534)
Supplement: Attachment — Submitted filename: Yoder Response to reviews.docx [file pone.0300534.s001.docx]

My comments are in blue. I believe all of the reviewers’ concerns and suggestions are addressed in the revised manuscript.

Lane Yoder

| **PLOS ONE <em@editorialmanager.com>** | Tue, Jun 22, 11:11 PM (9 hours ago) |  |    |
| --- | --- | --- | --- |
|  | | |  |

PONE-D-21-12646
Neural Flip-Flops I: Short-Term Memory
PLOS ONE

Dear Dr. Yoder,

Thank you for submitting your manuscript to PLOS ONE. After careful consideration, we have decided that your manuscript does not meet our criteria for publication and must therefore be rejected.

Specifically:

To support the claims made in the manuscript the author should develop and justify a neuronal model.  Development and investigation of such model would result in a different manuscript and would probably take  long time.

The manuscript has a neuron response model with justification in section 4.2.2.2 (line 319). The claims for the flip-flop model in Fig 4F are demonstrated by simulation using this neuron response model in section 4.3.3 (line 414). How this was done was clarified in section 3 (line 173).

A network simulation using a specific neuron response model can support network claims only for that response model. The manuscript goes substantially further by giving the minimal neuron properties that will produce the flip-flop claims (explained in section 3, lines 189-195).

I also recommend to check all references provided by the reviewers, since they have potentially made similar claims as this manuscript.

All but one of the reviewers’ references have been included in the revised manuscript, as explained below. These and other previous models are discussed in section 2.3 (line 106). Two of the most plausible models are discussed in some detail.

I am sorry that we cannot be more positive on this occasion, but hope that you appreciate the reasons for this decision.

Yours sincerely,

Gennady S. Cymbalyuk, Ph.D.
Academic Editor
PLOS ONE


[Note: HTML markup is below. Please do not edit.]

Reviewers' comments:

Reviewer's Responses to Questions

**Comments to the Author**

1. Is the manuscript technically sound, and do the data support the conclusions?

The manuscript must describe a technically sound piece of scientific research with data that supports the conclusions. Experiments must have been conducted rigorously, with appropriate controls, replication, and sample sizes. The conclusions must be drawn appropriately based on the data presented.

Reviewer #1: Yes

Reviewer #2: Partly

Reviewer #3: Partly

2. Has the statistical analysis been performed appropriately and rigorously?

Reviewer #1: Yes

Reviewer #2: Yes

Reviewer #3: Yes

3. Have the authors made all data underlying the findings in their manuscript fully available?

The PLOS Data policy

 requires authors to make all data underlying the findings described in their manuscript fully available without restriction, with rare exception (please refer to the Data Availability Statement in the manuscript PDF file). The data should be provided as part of the manuscript or its supporting information, or deposited to a public repository. For example, in addition to summary statistics, the data points behind means, medians and variance measures should be available. If there are restrictions on publicly sharing data—e.g. participant privacy or use of data from a third party—those must be specified.

Reviewer #1: Yes

Reviewer #2: Yes

Reviewer #3: Yes

4. Is the manuscript presented in an intelligible fashion and written in standard English?

PLOS ONE does not copyedit accepted manuscripts, so the language in submitted articles must be clear, correct, and unambiguous. Any typographical or grammatical errors should be corrected at revision, so please note any specific errors here.

Reviewer #1: Yes

Reviewer #2: Yes

Reviewer #3: Yes

5. Review Comments to the Author

Please use the space provided to explain your answers to the questions above. You may also include additional comments for the author, including concerns about dual publication, research ethics, or publication ethics. (Please upload your review as an attachment if it exceeds 20,000 characters)

Reviewer #1: The author proposes using AND-NOT gates to construct set reset flip-flops and short-term memory. The author claims that the AND-NOT gates act like neurons due to the capabilities of excitation and inhibition – and labels these as Neural Flip-Flops (NFFs). The author claims that a single neuron with one excitatory input and one inhibitory input, with signal strengths X and Y, can function as a logic primitive X AND NOT Y gate. I do not understand how this device can be labelled as a neural AND-NOT gate given that the device does not act like a neuron? Neurons are natural threshold oscillators and I cannot see any oscillatory behaviour in the circuits described in the paper at all. Paper [1], published in 2012, shows how threshold oscillator neurons can be connected together to form set reset flip-flops

Spike trains, which are referred to as “oscillations” in the 2012 paper, are discussed in section 4.2.1, lines 243-245 and 252, 253. The 2012 paper is discussed in section 2.3 (line 106).

and furthermore, it is shown that these circuits are remarkably noise resistant due to the oscillatory behaviour of the components.

The oscillatory behavior of spikes is irrelevant to noise resistance. The 2012 paper’s threshold model for a neuron response to excitatory input is illustrated in the figure below, with the strength of signals measured by the frequency of spikes. The threshold model is an extreme example of a sigmoid function and therefore satisfies the two minimum conditions required for noise reduction (inequalities 1 and 2, lines 306, 307). As pointed out in lines 293-294, a sigmoid response reduces moderate levels of additive noise in a binary input signal by producing an output that decreases a low input and increases a high input. The extremely robust noise resistance of the threshold response function is a result of the function’s extreme nonlinear behavior.

The authors’ claim on line 79 is incorrect – “apparently there is no other claim in the literature that a single neuron can function as a specific logic primitive based on the minimal neuron capabilities of excitation and inhibition.” In [1], it is clearly demonstrated that a single neuron can act as an AND gate, an XOR gate (both used in the paper to from a binary oscillator half-adder) and NOR and NAND gates (in SR flip-flops).

In the revision, the demonstration that a neuron can function as a specific neuron logic primitive was changed to “the first claim” rather than “the only claim” (lines 87-89).

NOR gates are used to build active high SR latches and NAND gates are used to build active low SR latches. The authors show that AND-NOT gates can also be used to build the two standard electronic flip-flops. The authors claim that ‘apparently’ no other papers produce the seven phenomena of short-term memory described using NFFs. Given that the author’s previous ‘apparently’ claim was false, I think that this needs further investigation.

This claim was omitted (lines 104, 105).

The circuit diagram for a single transistor AND-NOT gate is illustrated in Figure 3 and the authors shows how noise can be reduced. Again, as illustrated in [1], SR flip-flops constructed with threshold oscillators are highly resistant to noise and the noise does not have to be reduced in the components.

This apparently contradicts what the reviewer wrote above: “…these circuits are remarkably noise resistant due to the oscillatory behaviour of the components.”

The author refers to his earlier publications where it is shown that neural AND-NOT gates make up powerful fuzzy logic decoders whose architecture is radically different and more efficient than standard architectures.

Figure 5 shows the output of the NFF and that it is functioning correctly, however, the output is not functioning like neurons would?

If the 2012 paper’s threshold model were used for the simulation in Fig 5, all of the input noise in Fig 5 would be immediately suppressed. Both the neuron response model (Fig 2) that is used in the simulation and the two minimal response properties required for noise reduction (lines 306, 307) do not assume such ideal noise suppression.

The schematic for a neural memory bank is displayed in Figure 6.

I get the impression that these devices might be useful in fuzzy logic, however, this is not my area of expertise.


1. Borresen J, Lynch S (2012) Oscillatory threshold logic. PLoS ONE 7(11): e48498. doi:10.1371/journal.pone.0048498.

This reference was added.

Reviewer #2: The author presents a Neural Flip-Flop model of short-term memory. This model produces the main features of physiologically recorded activity underlying the storage of short-term memories. However, it is difficult to see how this model differs from other models of short-term memory, many of which are summarized in ref. 15 (Chaudhuri et al, 2016).

Previous models are discussed in section 2.3 (line 106).

Critically, the paper’s methods should be expanded to include the system of equations governing the network in Fig. 4d. This expansion would make it easier for readers to reproduce and evaluate the NFF model.

The operation of the network is clarified in section 3 (line 173). The simulation would be easy for anyone to reproduce.

Ideally, the author would make these comparisons to explicitly show how this model differs from the existing literature.

Except for the one NFF model discussed in section 2.3.3 (line 144), I haven’t found any previous model that can even be compared to the NFFs in Fig 4. This is made clear at the beginning of section 2.3.

Major:
The NFF model in Fig. 5 exhibits activity that resembles a discrete attractor model of short-term memory in which recurrent connections are combined with sigmoidal synapses to produce two stable states (i.e. Inagaki et al. 2019). These networks have been shown to robustly store information via mechanisms very similar to the NFF. I would appreciate if the author could compare and contrast the discrete attractor networks and the NFF more explicitly.

The Inagaki reference was added. What was said in section 2.3 (lines 107-115) applies to the discrete attractor model. It cannot be compared to the NNFs in the manuscript.

Page 6, The author describes ‘models based on synaptic strength changes’ as models of short-term memory. The referenced literature (refs 16-19) describes models of long-term memory on the hours to days timescale, and not the seconds time scale typically associated with short-term memory as in references 10-11. Therefore, none of these models capture the seven phenomena of short-term memory. However, the models described in references 13-15 can account for these fundamental experimental findings.

Yes. The confusion of the models of short- and long-term memory was clarified in section 2.3, lines 107-109.

Reviewer #3: The paper is an interesting read but as it make claims in theoretical neuroscience, it should be supported with neuron model simulations. I suggest that the proposed network should be implemented in a model as simple as integrate-and-fire so that the result is more convincing.

The NFF in Fig 4F is simulated in section 4.3.3 (line 414) using the simple neuron response model in section 4.2.2.2 (line 319). As mentioned above, the manuscript goes further than the simulation.

Also, although the paper contains a lot of historical references there is a lack of mention on recent work showing how NN can implement logic gates (see Vogels et al. 2005 for example https://www.jneurosci.org/content/25/46/10786 but there are many others).

Neural networks that implement logic gates are not really relevant to this paper, which is about neural flip-flops implemented with a single-neuron logic gate.

Also, on models of working memory the models based on synaptic changes like this one (Synaptic theory of working memory. G Mongillo, O Barak, M Tsodyks. Science 319 (5869)) should be discussed.

This reference was added.

6. PLOS authors have the option to publish the peer review history of their article (what does this mean?). If published, this will include your full peer review and any attached files.


If you choose “no”, your identity will remain anonymous but your review may still be made public.


**Do you want your identity to be public for this peer review?** For information about this choice, including consent withdrawal, please see our Privacy Policy.

Reviewer #1: **Yes:** Dr Stephen Lynch

Reviewer #2: **Yes:** Kayvon Daie

Reviewer #3: **Yes:** David Colliaux

[NOTE: If reviewer comments were submitted as an attachment file, they will be attached to this email and accessible via the submission site. Please log into your account, locate the manuscript record, and check for the action link "View Attachments". If this link does not appear, there are no attachment files.]

- - - - -
For journal use only: PONEDEC3

*In compliance with data protection regulations, you may request that we remove your personal registration details at any time. (Remove my information/details). Please contact the publication office if you have any questions.*

| <https://www.editorialmanager.com/pone/login.asp?a=r> | ReplyReply allForward |
| --- | --- |
