# Supplementary material for: Neural flip-flops I: Short-term memory
Source: PLoS One. 2024 Mar 15;19(3):e0300534. doi: 10.1371/journal.pone.0300534 (PMC10942071; doi:10.1371/journal.pone.0300534)
Supplement: Attachment — Submitted filename: Response to Reviewer.docx [file pone.0300534.s002.docx]

Neural Flip-Flops I: Short-Term Memory

Lane Yoder

My comments are in green.

Here are the main changes in the revision in response to the reviewer’s comments:

An important reference was added (#9). This June 2023 paper presents a general method of discovering how neurons are connected to process information. It is currently under review at PLOS One. The method was used to discover the neural flip-flops in this paper and the neuronal networks in references 1-6. This reference is discussed briefly at the end of the introduction.

The introduction to section 2.3 (Previous Models) was rewritten. A discussion of the characteristics necessary for a device to function as a memory mechanism was added.

The abstract was rewritten to clarify the paper’s contributions.

A reference (#33) that supports sigmoid neuron responses was added.

| **Date:** | Feb 28 2023 12:13PM |
| --- | --- |
| **To:** | "Lane Yoder" lyoder@hawaii.edu |
| **From:** | "PLOS ONE" plosone@plos.org |
| **Subject:** | PLOS ONE Decision: Revision required [PONE-D-22-30620] |

PONE-D-22-30620

Neural Flip-Flops I: Short-Term Memory

PLOS ONE

Dear Dr. Lane Yoder

Thank you for submitting your manuscript to PLOS ONE. After careful consideration, we feel that it has merit but does not fully meet PLOS ONE’s publication criteria as it currently stands. Therefore, we invite you to submit a revised version of the manuscript that addresses the points raised during the review process.


We look forward to receiving your revised manuscript.

Kind regards,

Wazir Muhammad
Academic Editor
PLOS ONE

Reviewers' comments:

Reviewer's Responses to Questions

**Comments to the Author**

1. Is the manuscript technically sound, and do the data support the conclusions?

The manuscript must describe a technically sound piece of scientific research with data that supports the conclusions. Experiments must have been conducted rigorously, with appropriate controls, replication, and sample sizes. The conclusions must be drawn appropriately based on the data presented.

Reviewer #1: Yes

2. Has the statistical analysis been performed appropriately and rigorously?

Reviewer #1: Yes

3. Have the authors made all data underlying the findings in their manuscript fully available?

The [PLOS Data policy](http://www.plosone.org/static/policies.action#sharing) requires authors to make all data underlying the findings described in their manuscript fully available without restriction, with rare exception (please refer to the Data Availability Statement in the manuscript PDF file). The data should be provided as part of the manuscript or its supporting information, or deposited to a public repository. For example, in addition to summary statistics, the data points behind means, medians and variance measures should be available. If there are restrictions on publicly sharing data—e.g. participant privacy or use of data from a third party—those must be specified.

Reviewer #1: Yes

4. Is the manuscript presented in an intelligible fashion and written in standard English?

PLOS ONE does not copyedit accepted manuscripts, so the language in submitted articles must be clear, correct, and unambiguous. Any typographical or grammatical errors should be corrected at revision, so please note any specific errors here.

Reviewer #1: No

I did not find any errors, and the reviewer did not note any specific errors here.

5. Review Comments to the Author

Please use the space provided to explain your answers to the questions above. You may also include additional comments for the author, including concerns about dual publication, research ethics, or publication ethics. (Please upload your review as an attachment if it exceeds 20,000 characters)

Reviewer #1: The manuscript entitled “Neural Flip-Flops I: Short-Term Memory” has been investigated in detail. The topic addressed in the manuscript is potentially interesting and the manuscript contains some practical meanings, however, there are some issues which should be addressed by the authors:

1) In the first place, I would encourage the authors to extend the abstract more with the key results. As it is, the abstract is a little thin and does not quite convey the interesting results that follow in the main paper. The "Abstract" section can be made much more impressive by highlighting your contributions. The contribution of the study should be explained simply and clearly.

The abstract was rewritten to clarify the paper’s contributions.

2) The readability and presentation of the study should be further improved. The paper suffers from language problems.

If the changes in the revised manuscript are not sufficient, specific examples of language problems would be helpful.

3) The importance of the design carried out in this manuscript can be explained better than other important studies published in this field. I recommend the authors to review other recently developed works.

The importance of the design is stated more clearly in the revised abstract, the end of the introduction, and the introduction to section 2.3. The results are discussed in section 5.

An important reference to recent work (reference #9, June 2023) was added. Reference #33 that supports sigmoid neuron responses was added. A few examples of other recent work are given in references 21-26, and two of the models are discussed in some detail in section 2.3. The problems with reviewing more such work are discussed in section 2.3.

4) “Discussion” section should be added in a more highlighting, argumentative way. The author should analysis the reason why the tested results is achieved.

Section 5 “Results” is a discussion section. As stated in the manuscript, the results are achieved because of design of the network with continuous feedback and the noise-reducing neuron responses to excitation and inhibition in inequalities 1 and 2 in section 4.2.2.1. It really is that simple, and there is nothing else to add. The results are illustrated in the simulation in Fig 5 and discussed in section 5.

5) The authors should clearly emphasize the contribution of the study. Please note that the up-to-date of references will contribute to the up-to-date of your manuscript. The study named- Crude oil time series prediction model based on LSTM network with chaotic Henry gas solubility optimization- can be used to explain the method in the study or to indicate the contribution in the “Introduction” section.

The contribution of the study is stated more clearly in the revised abstract, the end of the introduction, and the introduction to section 2.3.

An explanation of the method used to find the proposed networks was added with the discussion of reference #9 at the end of the introduction.

Thank you for the interesting suggestion, but it is not clear how LSTM can be related to the model in the paper.

6) How to set the parameters of proposed method for better performance?

It is not clear what parameters and performance this refers to, or how the performance of the model could be better. Inequalities 1 and 2 in section 4.2.2.1 are the only neuron properties needed for the results shown in the simulation in Fig 5.

7) It will be helpful to the readers if some discussions about insight of the main results are added as Remarks.

Section 5 “Results” is a discussion section. The main results were clarified in the abstract. Insight into the method of discovering the model was added with reference #9 at the end of the introduction.

This study may be proposed for publication if it is addressed in the specified problems.

Thank you very much. The comments are much appreciated.

6. PLOS authors have the option to publish the peer review history of their article ([what does this mean?](https://journals.plos.org/plosone/s/editorial-and-peer-review-process#loc-peer-review-history)). If published, this will include your full peer review and any attached files.


If you choose “no”, your identity will remain anonymous but your review may still be made public.


**Do you want your identity to be public for this peer review?** For information about this choice, including consent withdrawal, please see our [Privacy Policy](https://www.plos.org/privacy-policy).

Reviewer #1: No
