# Supplementary material for: Neural flip-flops I: Short-term memory
Source: PLoS One. 2024 Mar 15;19(3):e0300534. doi: 10.1371/journal.pone.0300534 (PMC10942071; doi:10.1371/journal.pone.0300534)
Supplement: Attachment — Submitted filename: Response to Reviewers.docx [file pone.0300534.s003.docx]

Neural Flip-Flops I: Short-Term Memory

Lane Yoder

My comments are in blue.

I want to thank the reviewers for their careful reviews and many thoughtful comments. They are much appreciated.

I haven’t included a revised manuscript because I have questions about what exactly needs to be revised.

| **PLOS ONE em@editorialmanager.com** [**via**](https://support.google.com/mail/answer/1311182?hl=en) **hawaii.edu** | Fri, Dec 15, 12:12 PM (18 hours ago) |  |      |
| --- | --- | --- | --- |
| to Lane  | | |  |

PONE-D-22-30620R1

Neural Flip-Flops I: Short-Term Memory

PLOS ONE

Dear Dr. Yoder,

Thank you for submitting your manuscript to PLOS ONE. After careful consideration, we feel that it has merit but does not fully meet PLOS ONE’s publication criteria as it currently stands. Therefore, we invite you to submit a revised version of the manuscript that addresses the points raised during the review process.

We look forward to receiving your revised manuscript.

Kind regards,

Sunder Ali Khowaja, Ph.D.
Academic Editor
PLOS ONE

**Additional Editor Comments:**

Dear Authors,

The reviewers have completed their review for this paper. One of the reviewers have shown concerns regarding the novelty and methodology of the proposed work. Authors are requested to prepare a thorough response for the next round of review.


[Note: HTML markup is below. Please do not edit.]

Reviewers' comments:

Reviewer's Responses to Questions

**Comments to the Author**

1. If the authors have adequately addressed your comments raised in a previous round of review and you feel that this manuscript is now acceptable for publication, you may indicate that here to bypass the “Comments to the Author” section, enter your conflict of interest statement in the “Confidential to Editor” section, and submit your "Accept" recommendation.

Reviewer #1: All comments have been addressed

Reviewer #2: (No Response)

Reviewer #3: (No Response)

2. Is the manuscript technically sound, and do the data support the conclusions?

The manuscript must describe a technically sound piece of scientific research with data that supports the conclusions. Experiments must have been conducted rigorously, with appropriate controls, replication, and sample sizes. The conclusions must be drawn appropriately based on the data presented.

Reviewer #1: Yes

Reviewer #2: Partly

Reviewer #3: Yes

3. Has the statistical analysis been performed appropriately and rigorously?

Reviewer #1: Yes

Reviewer #2: I Don't Know

Reviewer #3: Yes

4. Have the authors made all data underlying the findings in their manuscript fully available?

The [PLOS Data policy](https://urldefense.com/v3/__http:/www.plosone.org/static/policies.action*sharing__;Iw!!PvDODwlR4mBZyAb0!QCwtyboogQUuSvhnIB6VoLxlvFNnKBNzwNkHXRcpFu3ixj2_ufA6FcvBxYiU1tbU1a_o45x0hqgTW-BG$) requires authors to make all data underlying the findings described in their manuscript fully available without restriction, with rare exception (please refer to the Data Availability Statement in the manuscript PDF file). The data should be provided as part of the manuscript or its supporting information, or deposited to a public repository. For example, in addition to summary statistics, the data points behind means, medians and variance measures should be available. If there are restrictions on publicly sharing data—e.g. participant privacy or use of data from a third party—those must be specified.

Reviewer #1: Yes

Reviewer #2: No

Reviewer #3: Yes

5. Is the manuscript presented in an intelligible fashion and written in standard English?

PLOS ONE does not copyedit accepted manuscripts, so the language in submitted articles must be clear, correct, and unambiguous. Any typographical or grammatical errors should be corrected at revision, so please note any specific errors here.

Reviewer #1: Yes

Reviewer #2: Yes

Reviewer #3: Yes

6. Review Comments to the Author

Please use the space provided to explain your answers to the questions above. You may also include additional comments for the author, including concerns about dual publication, research ethics, or publication ethics. (Please upload your review as an attachment if it exceeds 20,000 characters)

Reviewer #1: The authors have discussed the issues I mentioned in detail. It is appropriate for the study to be accepted for publication.

Thank you for that opinion and for all of the comments in the first review.

Reviewer #2: The author has produced a simulation model based on the use of neuronal flipflops to describe several features of short-term memory. The concept is based on simple logic of 0/1 switches and the relative simplicity with which these symptoms can store information and provide a context for maintenance of information in short-term memory. I am reviewing this paper after it has already been reviewed by one reviewer who seems supportive of the overall paper itself but requested clarification.
As a neuroscientist rather than a computer scientist or mathematician, I generally find papers like this with no value in understanding brain function in any meaningful way. The system described here is completely artificial and really has no relevance to the way normal neurons interact. There is no synaptic pruning, no molecular changes, and as is well known, input to neurons is highly complex with thousands of inputs being provided. There are change in firing rates, firing patterns, oscillatory inputs that cause phase locking and spike-field coherence, etc that are all need to understand complex processes such as memory formation storage, recall and so on. Models such as this are potentially useful in trying to provide framework for understanding brain function but, in reality they are tools for the design of artificial neuro networks.

Yes, neurons have capabilities that are far more complex than just excitation and inhibition. But if a certain function can be accomplished simply, there is no compelling reason to believe that the actual mechanism necessarily uses more complex capabilities. Some biological designs do seem to be more complex than necessary, but that is far from the norm. On the contrary, if an extremely simple mechanism is possible, it seems more likely that natural selection might discover it first. This is essentially the principle of parsimony. See below for my comment on large numbers of inputs.

As I am not an expert in logic systems, it is difficult for me to comment on the rigor here, although based on my experience in computational neuroscience, I would say that the neuronal model here is exceptionally simplistic. I understand that this is one of the goals of the author- to indicate that a very simple system that can do powerful things- but this is by no means a new observation. Basic two and three layer neural networks that integrate inputs from input layers to produce a flip-fop like response have been able to perform such tasks for decades and continue to evolve in their complexity despite using fairly simple logic systems for decision making.

I have not found such models. In section 2.3.3, I reviewed in some detail the most plausible model I could find, but it had numerous problems. If the reviewer can provide a reference for an explicit model that performs the function of a flip-flop dynamically, i.e., in a few milliseconds, I will withdraw the paper. I would be disappointed of course, but I would very much appreciate knowing about such a model.

From a pure neuroscience point of view, I would argue that this paper has relatively low value, although from a computational and engineering perspective, this may not be the case and therefore, I would defer on this aspect.

I hope the reviewers would agree that a demonstration of how neurons can be connected to produce known brain phenomena should be considered valuable.

Regarding the paper’s substance, I would make a few comments. First, it is exceptionally long. It reads much more like a textbook than a research paper and a typical reader would get lost in the extensive descriptions of logic systems.

It seems that many neuroscientists appreciate some explanation of logic circuits, which apparently is not covered in typical neuroscience curricula. If the reviewer can recommend sections that can be omitted, I would be happy to do so.

This paper should be much more focused in the actual results, which in my opinion are very difficult to discern from discussion about the results, which are intermixed.

The actual results are intermixed with discussion about the results? I’m not clear on what this means. Can you elaborate? As far as I know, the paper only shows how certain results are derived but does not discuss them.

Second, I did not see a real example of a discreet case in which a memory is learned, recalled, etc. The author described case examples, but these seems to be presented more as a theoretical response. The reader has no idea what a specific memory test would look like.

A complete theory of memory is beyond the scope of the paper. The introduction notes that the neural decoders proposed in [2] can recall information that is held in NFFs, but other than that, the intention was only to show that neurons can be connected to form flip-flops. The mechanisms for providing inputs for setting and resetting NFFs, turning memory banks on and off, etc., are necessarily assumed to be outside of the networks.

Reviewer #3: The paper considers "neural flip-flops", bistable circuits of neurons, as possible devices for short-term memory in brain. Such flip-flops are thoroughly studied and widely used in electronics, so the only achievement of the paper is the assumption that similar systems can be relevant in neuroscience as well.

Yes, NFFs that accomplish the same functions as certain electronic flip-flops are the only result. The achievements are that this has apparently not been done before, and that the NFFs can produce the known phenomena of memory formation, retention, retrieval, termination, and errors.

There is no surprise that one can come up with a simple circuit which demonstrates bistable behaviour,

Yes, it is not surprising. The only surprise is that it apparently has not been done before.

but I highly doubt that circuits similar to NFFs are used in working memory of real brains. The most obvious reason is that at least one neuron of the NFF is always ON which is very expensive in terms of power.

The NFF models each have a neuron that fires at a high rate while information is stored in memory. This is consistent with experimental evidence. The neural memory bank in section 4.3.4 shows how all of the NFFs used for the stored information can be inactivated when the information is no longer stored, which is also consistent with experimental evidence.

The other reason is that the brain functions barely rely on such small circuits due to low reliability of single neurons.

The brain could have logic circuits that operate in the same way as NFFs in principle if not in the details. The need for redundancies and other error-correcting mechanisms due to the low reliability of single neurons is one reason for requiring many more neurons for any network without affecting the underlying method.

After all, such a simple model does not suggest any explanations for the key characteristics of the working memory: capacity and duration, which can be unlimited in a population of NFFs.

I may not understand this comment. The capacity, i.e., the number of neurons devoted to NFFs, is potentially large but obviously not unlimited. The capacity could be further limited by natural selection, based on need. The duration can be limited by turning parts of the memory bank off, as indicated in section 4.3.4.

For the above reasons I do not see much scientific value in the present study and can not support its publication.

I hope this opinion can be changed.

Thank you.

Lane Yoder

7. PLOS authors have the option to publish the peer review history of their article ([what does this mean?](https://urldefense.com/v3/__https:/journals.plos.org/plosone/s/editorial-and-peer-review-process*loc-peer-review-history__;Iw!!PvDODwlR4mBZyAb0!QCwtyboogQUuSvhnIB6VoLxlvFNnKBNzwNkHXRcpFu3ixj2_ufA6FcvBxYiU1tbU1a_o45x0hipiF3VP$)). If published, this will include your full peer review and any attached files.


If you choose “no”, your identity will remain anonymous but your review may still be made public.


**Do you want your identity to be public for this peer review?** For information about this choice, including consent withdrawal, please see our [Privacy Policy](https://urldefense.com/v3/__https:/www.plos.org/privacy-policy__;!!PvDODwlR4mBZyAb0!QCwtyboogQUuSvhnIB6VoLxlvFNnKBNzwNkHXRcpFu3ixj2_ufA6FcvBxYiU1tbU1a_o45x0hv1mabDM$).

Reviewer #1: No

Reviewer #2: No

Reviewer #3: No
