# Supplementary material for: Neural flip-flops I: Short-term memory
Source: PLoS One. 2024 Mar 15;19(3):e0300534. doi: 10.1371/journal.pone.0300534 (PMC10942071; doi:10.1371/journal.pone.0300534)
Supplement: Attachment — Submitted filename: Response to reviewers.docx [file pone.0300534.s004.docx]

I would still like the Author to reply on my last comment regarding the capacity and duratino of the working memory. It is known that it can store a limited number of items (about 7) for a limited amount of time (tens of seconds). How can these properties be explained by the suggested model?

Thank you for this. I didn’t quite understand the question in the previous review. The following subsection was added in the revision on page 26.

**5.3. Limited capacity and duration of short-term memory**

Compared to long-term memory, short-term memory can store only a small amount of information and only for a short time. The NFF model may provide at least a partial explanation for these limitations.

Short-term memory is short not only in duration but also in formation (a few milliseconds). Fast memory formation is an obvious biological advantage, even necessary for many mundane functions. That advantage could well have been the selective pressure that led to short-term memory. Speed means the mechanism must be dynamic in the sense that the only changes are the strengths of the neurons’ signals.

Long-term memory is static. Memory models typically involve structural changes, such as neurogenesis, synaptogenesis, or pruning, or changes in synaptic strength or the strength of action potentials. Such changes may provide the large capacity and robust durability of long-term memory, but the changes are too slow for the fast formation of short-term memory.

Dynamic operation has the advantage of speed, but there are tradeoffs. First, NFFs’ dynamic operation is expensive in energy use because at least one neuron in each NFF is highly active continuously while information is held in memory. This NFF activity is consistent with empirical evidence in short-term memory [14-16]. Second, the dynamic operation makes NFFs’ stored information volatile. The state of an NFF can be inverted by temporary high additive noise in a set or reset input, a temporary loss of continuous energy input, or a temporary loss of a continuous high signal input. Neurons are notoriously unreliable in these aspects, which would have a detrimental effect on the duration of memory implemented with NFFs. The cessation of the memory neuron activity before an error is made in a memory test is consistent with empirical evidence in short-term memory [14-16].

The memory bank in Fig 6 shows that many NFFs can be deactivated when information stored in short-term memory is no longer needed. This energy-saving function shortens the duration of short-term memory. The cessation of the memory neuron activity when information is no longer held in memory after a memory test is consistent with empirical evidence in short-term memory [14-16].

The capacity of short-term memory could be affected by its limited duration. The number of neurons devoted to short-term memory may be limited by the brain’s ability to use information before the memory decays or becomes useless. If the prefrontal cortex cannot process large amounts of information conveyed by the senses at a sufficiently fast rate, there would be no reason to store that much information.

The capacity and duration of short-term memory could be affected by the difficulty in forming links. Long-term memory evidently relies on links for memory formation and retrieval. A name can be recalled from information associated with it. An extraordinary example of linkage is the number of songs that can be learned and recalled for decades, with seemingly effortless memory formation and recollection. A song’s lyrics and melody are each connected linearly, and the two are connected to each other in parallel.

Short-term memory may not involve information that has such connections. Short-term memory often involves pseudorandom information (e.g., a phone number) that has no apparent possibility for a link. Arduous mnemonic techniques for linking unfamiliar items to familiar objects and places, such as “memory palaces,” can increase the capacity of short-term memory somewhat, but such techniques fall far short of the capacity of long-term memory and have little or no effect on duration.

Other, unknown differences between long- and short-term memory may affect capacity or duration, such as how information is encoded in memory and how it is decoded.
